# Developing Multipurpose Reproductive Health Technologies: An Integrated Strategy

**DOI:** 10.1155/2013/790154

**Published:** 2013-02-28

**Authors:** P. F. Harrison, A. Hemmerling, J. Romano, K. J. Whaley, B. Young Holt

**Affiliations:** ^1^Coalition for Advancing Multipurpose Innovations (CAMI)/Public Health Institute, USA; ^2^AVAC, Global Advocacy for HIV Prevention, New York, USA; ^3^University of California, 50 Beale Street, Suite 1200, San Francisco, CA 94105, USA; ^4^NWJ Group, LLC, USA; ^5^Mapp Biopharmaceutical, San Diego, USA

## Abstract

Women worldwide confront two frequently concurrent reproductive health challenges: the need for contraception and for protection from sexually transmitted infections, importantly HIV/AIDS. While conception and infection share the same anatomical site and mode of transmission, there are no reproductive health technologies to date that *simultaneously* address that reality. Relevant available technologies are either contraceptive or anti-infective, are limited in number, and require different modes of administration and management. These “single-indication” technologies do not therefore fully respond to what is a substantial reproductive health need intimately linked to pivotal events in many women's lives. This paper reviews an integrated attempt to develop multipurpose prevention technologies—“MPTs”—products explicitly designed to *simultaneously* address the need for both contraception and protection from sexually transmitted infections. It describes an innovative and iterative MPT product development strategy with the following components: identifying different needs for such technologies and global variations in reproductive health priorities, defining “Target Product Profiles” as the framework for a research and development “roadmap,” collating an integrated MPT pipeline and characterizing significant pipeline gaps, exploring anticipated regulatory requirements, prioritizing candidates for problem-solving and resource investments, and implementing an ancillary advocacy agenda to support this breadth of effort.

## 1. Introduction

The combined burden of maternal and infant mortality and morbidity produced by unintended pregnancies and sexually transmitted infections—individually and as a consequence of their multiple interactions—is compelling in its volume, extent, and complexity. For an array of behavioral, biological, physiological, and sociocultural and political reasons, most of that burden falls on women in developing countries. In those countries, of the 80 million unintended pregnancies estimated for 2012, 63 million will occur among the 222 million women defined as having an “unmet need” for modern contraception [1]. Those unintended pregnancies will, in turn, result in 30 million unplanned births; 10 million miscarriages, including stillbirths; and 40 million abortions, of which one-third to one-half will be unsafe. Women aged 15–19 are at particular risk of these events [2].

Sexually transmitted infections (STIs) further compound these burdens, with which they are relentlessly intertwined. The World Health Organization estimates that 448 million new cases of the curable STIs (trichomoniasis, chlamydia, gonorrhea, and syphilis) occur annually in adults aged 15–49 years [3]. Cases of the major incurable viral diseases—genital herpes (HSV-2), human papillomavirus (HPV), and HIV-1—account for an even greater burden of both morbidity and mortality. The estimated number of people aging 15–49 years living with HSV-2 worldwide in 2003 was 536 million, with overall prevalence higher in women than in men [4]. Each year an estimated 493,000 women are diagnosed with cervical cancer, largely attributable to HPV infection; over 273,000 die from the disease, 234,000 of those in developing regions [5]. Lastly, there is HIV, with 2.7 million new infections in 2010, of which, in many regions and subpopulations, women account for over half.

Over 20 years ago, a seminal review described the relationship between the “classical” STIs and HIV-1 as an essentially lethal “epidemiologic synergy” [6]. The authors presented persuasive evidence that both ulcerative and nonulcerative STIs significantly promoted HIV transmission by augmenting HIV infectiousness and susceptibility, concluding that STI treatment should therefore be an essential component of HIV prevention strategies. Yet, while subsequent studies continued to document and elucidate those relationships, interest in addressing the relationship between STIs and HIV-1 waned, primarily because it has “proven nearly impossible to reduce the spread of HIV-1 through directed or empirical treatment of STDs” [7].

Addressing contraception and STI prevention in meaningfully coordinated fashion has had limited success, even given potential cost savings [8]. Typically, women must seek care for contraception and HIV prevention from separate health facilities and different providers, and examples of truly functional integration of HIV, STI, and family planning services remain rare. Such efforts can be organizationally and/or financially difficult to implement and HIV-associated stigma may act as an additional barrier. 

The premise of the work reported here is that multipurpose prevention technologies—“MPTs”—addressing more than a single reproductive health indication with a single administration would offer an additional route to integrated reproductive health. Potential components for such products exist, some already commercially available; new components and formulations are also likely to be required. MPTs could comprise combinations of HIV prevention technologies with agents having contraceptive activity; available contraceptives and agents active against HIV; single drugs targeting more than one indication; totally new drug combinations or combinations of drugs and devices; and/or multi-indication vaccines. 

## 2. Materials and Methods

This paper describes an innovative process designed to advance the development of MPTs through systematic, iterative consideration of, first, the key components of a standard product development pathway and, second, different product requirements in different user populations. The organization of the account that follows below responds to the general categories proposed for the COREQ, the checklist of consolidated criteria for reporting qualitative research [9].

### 2.1. Research Teams

Advancing the MPT concept was expected to be scientifically, technologically, and practically challenging. Mobilization of the scientific and financial resources required for MPT development would demand a sound evidence-based argument for the need for MPTs and their plausibility as a product category, plus an integrated mix of expertise and advocacy. To that end, the Initiative for Multipurpose Prevention Technologies for Reproductive Health (IMPT) was founded in 2009 as a global coalition of multidisciplinary and multinational stakeholders, scientists, policy-makers, advocates, donors, and product developers [57]. Housed at CAMI (Coalition Advancing Multipurpose Innovations) in California, USA, the IMPT was organized as a nonaligned convener for affiliates and as an umbrella for working groups and teams with specific research and advocacy responsibilities. 

The activities of the Initiative and its colleagues focus in three areas:

1. defining an integrated MPT product pipeline and scientific agenda, as guidance for donors, product developers, regulators, and advocates about MPT scientific priorities and needs;
2. exploring the associated regulatory pathways and anticipated needs around delivery of and access to such products;
3. designing and implementing a strategy for communication, advocacy, and outreach to raise global awareness around MPTs as a prospective public health product.

**Figure 1:**
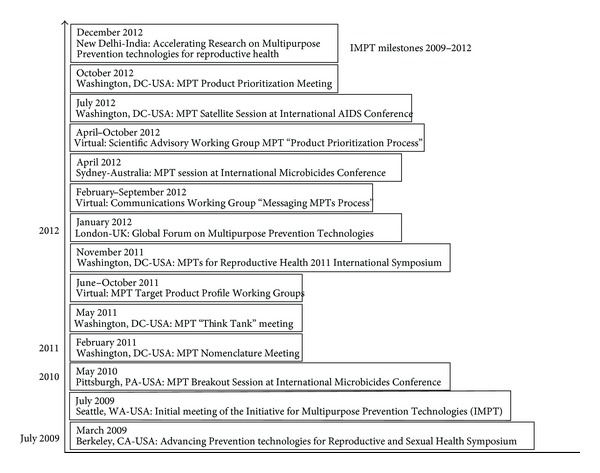
MPT Strategic Milestones 2009–2012.

The emphasis in this paper is on the first two of these activities and the teams formed sequentially for their implementation: the Think Tank, Drug-Drug/Drug-Device Working Group, Multipurpose Reproductive Health Vaccine Working Group, and Scientific Agenda Working Group (SAWG). The CAMI Advisory Committee and Management Group was led by the authors of this paper and was responsible for the overall conceptual guidance and management of the entire process.  Figure 1 presents the chronology of team formation and key MPT process-related activities; Table 1 in the section on study design summarizes the objectives and methodology used by each team and their contributions to the strategy process. 

## 3. Study Design

### 3.1. Theoretical Framework

The novelty, breadth, and complexity of the MPT concept required a comparably broad and complex methodology. The Advisory Group opted for an iterative research and advocacy strategy comprising focused consultations, surveys, qualitative data-gathering, and pipeline analysis, all informed by input from scientists, product developers, representatives from relevant geographic regions, and donors. The strategy objectives were to assess the scientific feasibility of the MPT concept, develop an “MPT Target Product Profile,” and define and prioritize a scientific agenda for MPT research and development that would inform MPT investment, policy, and advocacy.

Assessing the potential of each MPT candidate according to the desired TPP was expected to (1) identify the nature and magnitude of required resources for advancing the most promising pipeline candidates, (2) flag unproductive redundancies in the overall pipeline, and (3) avoid duplication of effort or development of products that fail to meet the minimum requirements of the TPP. The intended output was a “road map” permitting researchers, policy-makers, and donors to make decisions about next research steps and investments along the entirety of the MPT research and development pathway and identify the potential for efficiencies that might be achieved by strategic collaborations among researchers and developers. Such a review process and the resulting road map was expected to support the best alignment of technologically feasible MPTs with products identified as “ideal” by women and health care providers in regions and populations that would most benefit from multipurpose prevention technologies intended to foster and support improved overall reproductive health. 

#### 3.1.1. MPT Target Product Profile

Adoption of this methodology as a major organizing concept for the MPT strategy emerged from the deliberations of the May 2011 MPT Think Tank (Table 1), against a background of increasing interest among major health and development donors in Target Product Profile (TPP) approaches. While variously defined and applied by the pharmaceutical industry and the US Food and Drug Administration, the TPP is a goal-oriented template for assessing and prioritizing candidate biomedical products in terms of their development progress and potential and, in some cases, market prospects and likely impact [56, 58]. Each MPT strategy team was asked to adapt that basic TPP concept by selecting the attributes, parameters, and associated criteria for MPT products that would offer the highest potential public health impact for their putative user populations, responsiveness to the unmet needs of those populations, and satisfaction of the major MPT objective: contraception and prevention of HIV and non-HIV STIs simultaneously delivered in a variety of modalities.

#### 3.1.2. Product Prioritization

Construction of Target Product Profiles for MPTs involved successive prioritizations of their main elements: primary indications (HIV prevention/contraception, HIV/STI prevention, and STI prevention/contraception; routes of administration and dosage forms; product attributes and parameters (e.g., stability, infrastructure needs, reversibility); and safety, efficacy, and potential for uptake. These individual prioritizations would then contribute to a “consensus TPP” that could shape general development priorities and fundamental design targets that would, in turn, guide funder investment prioritization and developer R&D focus. The process would include compilation of a comprehensive list of candidate MPT-related products and product components, followed by interrelated evaluations for development feasibility, number of candidates per product type, “fit” with general TPP findings, and input from the contraceptive field. This work would be implemented by the Scientific Agenda Working Group (SAWG) and its Product Prioritization subgroup; a similar process would occur in the Multipurpose Reproductive Health Vaccine Working Group. 

#### 3.1.3. Understanding Regional Needs and Priorities for MPT Development

MPT need and demand would necessarily be affected by the fact that global unmet need and demand for modern contraception are quite variable, as are the epidemiological profiles of HIV and STI incidence, prevalence, and contribution to overall burdens on women's health. Thus the MPT prioritization process would have to take into account the types of target populations in specific geographic regions most likely to benefit from MPTs and be interested in using them. 

While the MPT strategy had steadily incorporated perspectives from those regions, the principal methodological contribution to this component was the January 2012 Global Forum on MPTs, which convened 60 participants from Africa, the Caribbean, China, Europe, India, United Kingdom, and the United States to elicit international multisectoral input into draft TPPs, extend consideration of critical path for regulatory approval of MPTs beyond the US Food and Drug Administration (USFDA) to other regional regulatory authorities, encourage global perspective and international support for MPTs, and seek consensus on next steps. The results of this process component are summarized below in Table 5.

## 4. Results: Findings and Analysis

### 4.1. Primary Indications for MPT Drug-Drug and Drug-Device Combinations

Across working groups and respondents to different data-gathering approaches, consensus emerged that the most critical parameter in the construction of an MPT Target Product Profile was the *combination of indications* to be met by a given product, that is, contraception, HIV prevention, and/or prevention of non-HIV-STIs. Overall, the combination of HIV prevention and contraception was assigned the highest priority, followed closely by HIV + HSV. Non-HIV STIs were variously prioritized in terms of relevance for HIV transmission, technical feasibility, epidemiological burden, and effectiveness of available treatments. 

Variability among informant populations did produce noteworthy differences in rankings. Comparison of findings from the surveys among US and African reproductive health care providers (Table 1) found that 66 percent of African providers ranked unintended pregnancy + HIV as of highest priority, while the same percentage of US providers ranked unintended pregnancy + non-HIV STIs as the highest-priority target indication. HPV was ranked as the highest-priority non-HIV STI by both survey populations (75 percent and 68 percent of African and US providers, resp.). While response volumes from China and India were not high, the combination of contraception + non-HIV STIs appeared to command the most interest as MPT candidates for those markets.

### 4.2. TPP Parameters for Prioritizing MPT Development

The consensus Target Product Profile for MPTs comprised a defined set of parameters with associated “preferred” and “minimally acceptable” criteria that formed the architecture for determining what must matter most for MPT development once the highest-priority indication has been determined. Those assigned priority through the methods described in the preceding section appear above in Table 2. Several of these attributes received intense scrutiny and thus merit additional comment.

*Dosage Forms.* Given broad consensus that a crucial arbiter of efficacy for any MPT will be adherence to correct product use, it was not surprising that sustained-release devices, importantly intravaginal rings (IVR), were identified as the highest-priority dosage form. The rationale for IVR as a preferred delivery system was that such technologies, which could be user-inserted and designed for at least 30 days of efficacy, offered potential for greater adherence compared to other user-administered systems. IVRs are reversible, may impose less of a burden on health systems and, depending on drug activity, might also mitigate some of the side effects associated with oral administration and correspondingly greater systemic exposure. Again, however, there was variation across survey populations. US providers preferred oral dosage forms, while African providers leaned toward a “suite” of several dosage forms as offering greater potential for acceptability and use, and ranked injection and sustained-release devices slightly higher than others.

*Efficacy Targets.* There was consensus that MPT components for HIV prevention should meet a minimum requirement of 40–50% reduction in risk, preferably at least 80% with perfect use and 60% with typical use. Contraceptive MPT components should be no less effective than currently available products and an efficacy minimum of at least 40% was the target for prevention of non-HIV STIs.

*Product Attributes*. Most specific attributes were identified within the context of safety, efficacy, and other factors, with a relatively long shelf life (36 months) and storage at high temperature (40°C) as the most consistently-supported priorities.

*Side Effects*. The general view was that these would need to be assessed in the context of the overall safety and anticipated efficacy of the MPT under consideration, but should be “no worse than individual indication products,” for example, currently available contraceptives.

*Other Parameters.* Another group of “non-TPP parameters” emerged in the research and review process as issues requiring further discussion with respect to their importance for different potential user populations. Those were research entity, resupply infrastructure, access to testing/monitoring, cold chain storage (if needed), time to development for compounds, potential drug interactions, mechanism of action established in other products (e.g.,Truvada, NuvaRing), novelty of mechanism of action and enhancement of pipeline diversity, pipeline redundancy, potential for drug resistance, potential for discreet use, influence on sexual experience, incidence/prevalence in target population and overall burden of disease, and few or no existing or readily available treatment options.

### 4.3. Multipurpose Reproductive Health (RH) Vaccine Working Group

The Multipurpose RH Vaccine Working Group's “Request for Concepts” elicited 13 submissions and/or comments, almost all based on active immunization (Table 3) and responsive to the Target Product Profile developed by this group (Table 4). Two additional concepts were based on passive immunization [59] and adenovirus vectored antibodies [60, 61] and one submission was focused on product development strategies. In general, it was recognized that advances in mucosal vaccinology were crucial to advancement of these concepts [62].

### 4.4. Understanding Regional Needs and Priorities for MPT Development

The information that has accumulated with respect to regional priorities for MPTs has accelerated in volume, coverage and, with the refinement of the Target Product Profiles, its relevance to MPT development writ large. The January 2012 Global Forum on MPTs hosted by the Wellcome Trust was explicitly designed to elicit international multisectoral input into the draft TPPs, extend consideration of the critical path for regulatory approval of MPTs beyond the perspectives of the USFDA to include the views of representatives from other regulatory authorities; encourage a global perspective and international support for this Initiative and seek consensus on next steps, and identify the types of target populations in specific geographic regions most likely to benefit from MPTs. Table 5 summarizes the extensive output of that vital consultation and background material provided by its participants.

#### 4.4.1. The MPT Pipeline

Extensive research by the Scientific Agenda Working Group (SAWG) and colleagues also recently generated the first comprehensive list of all known potential MPT candidate products and components, concepts, relevant technology platforms, and delivery systems responsive to the major MPT indications. The drug candidates in this listing were then subcategorized by mechanism of action, chemical class; product candidates were organized according to dosage form and stage of development. In addition to yielding a summary set of MPT product priorities, this review and analysis process revealed certain imbalances in the R&D efforts being invested in different MPT product and component types.

#### 4.4.2. Pipeline Prioritization and Gap Identification

*Priorities.* The exercise to prioritize MPT candidate drugs and products identified specific active pharmaceutical ingredients (API) and product configurations appropriate for timely and effective development of MPT products. In light of the priority indications of HIV and pregnancy prevention, MPTs that involve small organic molecule antiretroviral (ARV) agents and hormonal contraceptives were prioritized. The lack of candidate STI prevention options did not allow for specific prioritization for this indication (see in what follows). Further, it was recognized that a suite of MPT product configurations would be necessary to achieve maximum public health impact. Specifically, vaginal rings, long acting injectables, and alternative on-demand formulations are all defined as priority configurations for MPT products.

*Gaps.* A range of gaps were identified in the course of the prioritization exercise. Specifically, it was noted in the following:

- There is a lack of alternatives to reverse transcriptase inhibitor (RTI) antiretrovirals (ARV) for the HIV indication.
- Sufficient understanding of the potential relationship between specific forms of hormonal contraception (e.g., injectable DMPA) and increased risk of HIV transmission is lacking.
- Viable, pathogen-specific options for the non-HIV STI indication for potential MPTs are unavailable.
- There are insufficient data on acceptability, use, and uptake of intravaginal rings.
- Too few options for long-acting injectable delivery modalities are in development.
- Insufficient knowledge about the safety of intermittent use of ARVs and other anti-infectives is a risk for on-demand product options in general.
- Limited non-hormonal-contraception and STI-prevention options exist.
- Definitive social-behavioral science to support all product options is limited.

*Needs.* The analysis also generated a short list of early-stage development candidate categories meriting pursuit for possible longer-term development:

- STI-specific APIs;
- non-ARV-based HIV prevention;
- lactobacillus-based products;
- nonhormonal contraceptives;
- novel on-demand product configurations.

*Process Priorities.* The product prioritization exercise also generated a set of “process priorities,” the absence of which could hinder the MPT effort in the longer term. The key process priority is the need for coordination across donor investments, sponsor development, and program management. This, in general, has been seen as desirable but often absent; however, current resource limitations and the complexities around MPTs dictate the urgency of

- consensus on priority products, gaps, and development strategies,
- a coordinated approach to identify single-lead products for each priority MPT product type,
- pooling of capacity, capability, expertise, and other resources between viable development entities interested in MPT products,
- coordinated investment and collaborative/partnered development management,
- early and proactive engagement of regulatory authorities, supported by TPP templates specific to product types.

## 5. Discussion and Conclusions

### 5.1. The State of MPT Research and Development

The purpose of the MPT Scientific Agenda Working Group activities is to inform and provide guidance for donors, product developers, and regulators about MPT priorities and investment needs. It adopted an iterative strategy of steps, feedback loops, adjustments to its own received wisdom and that of others, and an expanding circle of engagement that could inform the process but not cripple it. 

The IMPT will continue its iterative process of sharing the priorities identified and associated recommendations, particularly in the area of drug/drug and drug/device combinations, with an expanded range of stakeholders, including regional experts, sociobehavioral scientists, clinicians, and manufacturers. It will also continue to monitor the MPT pipeline and support coordinated donor and developer engagement. As the MPT field advances, MPT product priorities will evolve and expand as the realization of multipurpose reproductive health vaccines, with a longer time line, also proceeds. Both MPT categories are believed to offer considerable potential for innovation, public health impact, and a sizable market in both the developed and developing worlds. 

Some critical building blocks are already in place for drug-drug and drug-device MPTs. There are putative MPT components in products long approved for single indications and in contraceptive products already commercially available in multiple configurations. Drugs for treatment of HIV and STI are available, though in some cases imperfect and problematic, and infectious disease prophylaxis is established for some indications. HIV prevention of mother-to-child transmission (PMTCT) is proving effective and results from late-stage trials of Pre-Exposure Prophylaxis of vaginal and oral products suggest that HIV prevention is, with some critical questions to be asked and answered, within reach [63].

A few MPT candidates have completed discovery and are in ongoing development:

- an intravaginal ring that continuously releases tenofovir and levonorgestrel from separate ring segments over a period of 90 days, for contraception and HIV prevention [64];
- a gel combining MIV-150, zinc acetate, and carrageenan, with combined activity against HIV and HSV [65];
- a vaginal ring releasing dapivirine and a hormonal contraceptive over 60 days for contraception and HIV prevention [66, 67];
- reformulated tenofovir gel is being studied in conjunction with the existing SILCS diaphragm as a combined barrier contraceptive, adding sperm immobilizing agents and antiviral chemical protection against HIV and HSV [64].

### 5.2. Allied Efforts

The structure of the Initiative for Multipurpose Prevention Technologies and the work of its colleagues were explicitly designed to take into account that simply having an MPT pipeline and prioritizing R&D investments would not be sufficient to getting an actual MPT on the market and into the hands of users. Thus, while the activities of the Scientific Agenda Working Group (SAWG) are the focus of this paper, that work could not have evolved nor can it continue without a range of support, importantly including financial resources. 

Thus, the Initiative has, through a cross disciplinary approach, implemented a series of activities to support the emerging MPT field and the Scientific Agenda derived from the work of the SAWG. Among those activities are working groups charged with Communications, Advocacy and Outreach and with MPT Acceptability and Access. Both teams aim to increase global awareness and support for MPT development among scientists, donors, policy-makers, regulators, health care providers, and advocates. The Communications, Advocacy and Outreach Working Group has identified and convened regional experts and affiliates in a number of different countries with high unmet need for MPTs (e.g., China, India, Jamaica, Kenya, South Africa, and Tanzania) to help shape the scientific agenda, ensure that the MPTs that are developed will be socially and culturally appropriate and craft messages to raise awareness and support for product research and development. The allied MPT Acceptability and Access Working Group aims to ensure that MPT products will be accessible and affordable for those with highest unmet need, through attention to potential regulatory requirements for MPT products and exploration of the most promising delivery pathways for MPTs in different global regions. 

## 6. Conclusions

The road to even the first MPT product will not be smooth. That became clear as the MPT Prioritization Process fulfilled its mandate to highlight key challenges, gaps, and needs if MPTs are to be realized efficiently and with reasonable speed. Different chemical compounds may require different conditions for formulation and release and the human vaginal environment is difficult to mimic accurately in *in vitro* laboratory or animal experiments. Drug interactions between concurrently released compounds could impact product efficacy and safety. The impact of hormonal contraception on HIV transmission has recently risen to a global level of concern and awaits clarification. There is much to be known about *in situ* placement of vaginal devices in terms of safety, drug uptake, and distribution, and timely efficacy with respect to prevention. Since women's needs vary in different regions of the world and throughout their lives, a single MPT will not be fully responsive and a “suite” of MPT products will be critical for these new technologies to have optimum public health impact.

Because the prospective user populations for many MPTs reside in a range of economic and epidemiologic settings, review of MPTs will require experts from different fields and collaboration among international regulatory and national health authorities. While the preferential use of already approved drugs and devices as MPT compounds may save time and resources in the navigation of regulatory requirements and although FDA approval for drugs and devices can facilitate and accelerate drug approvals in other countries, a comprehensive drug development strategy must nonetheless include regulatory requirements for all target markets. 

There are also the linked questions of cost and effectiveness. There is solid evidence for substantial cost savings to be derived from responding to unmet needs for modern contraceptives [2]. Simple modeling exercises indicate that the potential of MPTs to increase product adherence could lead to meaningful positive economic benefits [68]. 

Advancement of scientific research and public health technologies, particularly innovative technologies for the developing world, has traditionally confronted constraints: insufficient funding, regulatory barriers, private industry perceptions that products designed for the developing world offer scant profit, numerous impediments to product availability, and, sometimes, lack of fit between the technology involved and the population it was meant to benefit. All these constraints have surfaced repeatedly and in some ways uniquely in reproductive health, owing to deep-rooted cultural, political, and socioeconomic factors. 

In sum, developing a menu of prevention technologies, indeed even the first such technology, will take years; the perseverance of scientists, donors, and advocates; and the capacity to deal with the inevitable failures inherent in drug development. However, emerging from the most recent conversations hosted by the SAWG was agreement on the requirement to “think big” and resist temptations to settling on refinements of what is already in the pipeline, since multipurpose technologies that simultaneously address two primary reproductive health needs for women worldwide justify the imagination, skills, and sheer grit that will be required for their realization.

## Figures and Tables

**Table 1:** Study design components.

Teams and associated activities*	Tasks and deliverables	Data collection approach	Participants	Process contributions (summary conclusions/material outputs)
CAMI Advisory Committee and Core Management Group	Overall design and management of strategic process	Regular telephonic and internet consultation, document preparation, and review	(i) Core strategy management group (*N* = 6) (ii) Entire CAMI Advisory Committee (*N* = 24)	Series of meeting reports, circulation of survey findings, web posting of presentations, conference convening

Think Tank*	Answer 2 questions: (i) Is MPC concept scientifically feasible? (ii) If so, what is most logical and effective way to organize and prioritize the scientific agenda for MPT R&D? (Results to be discussed at International MPT Symposium, November 2011)	Document review and consultation (May 2011) (i) Review of ideal characteristics for populations most likely to benefit from MPTs (ii) Review of pipeline of relevant technologies (iii) Preliminary definition of research needs, gaps, obstacles for each MPT component	28 representatives from businesses, foundations, universities, nonprofit organizations, US government agencies (USAID, NIH, FDA)	Conclusions: (i) MPTs deemed feasible, though scientifically challenging (ii) Recommended adoption of Target Product Profile methodology for MPTs (iii) Agreed to form two teams to develop specific TPPs for (a) combination drug and drug/device MPTs and (b) multipurpose vaccines

Drug-Drug and Drug-Device Working Group	Implement strategy to: (i) Select and refine TPP critical attributes and appropriate parameter ranges for high-impact MPTs in these product categories (ii) Expand understanding of regional needs and priorities for MPT development	“Snowball” series of surveys, ePolls, qualitative interviews, consultations, invited presentations, consultations, and reviews of successive iterations of TPP parameters and criteria (March 2011–January 2012)	Key populations: reproductive health and HIV research experts and advocates from Asia, Africa, Europe, United States, including (i) 593 US health care providers (Association for Reproductive Health Professionals (ARHP) 2011 Conference) (ii) 289 African health care providers (International Family Planning (ICFP) 2011 Conference) (iii) ~120 participants, MPT 2011 Symposium	Consensus derived from each survey analyzed to construct consensus TPP for presentation, discussion, feedback from participants in International MPT Symposium (November 2011, Washington, DC) and >60 participants at Global Forum on Multipurpose Prevention Technologies (London, UK, January 2012)* for discussion

Multipurpose Vaccine Working Group	(i) Elicit ideas for multipurpose reproductive health vaccines (ii) Develop consensus Target Product Profile (iii) Discuss timeline for MPT vaccine development	(i) “Request for MPT Concepts” formulated, reviewed, emitted (ii) Teleconference process to develop Target Product Profile for MPT reproductive health vaccines	*N* = 15 MPT vaccine researchers and potential developers	13 submissions received based on active immunization, passive immunization, adenovirus-vectored antibodies, and MPT vaccine development strategies

Scientific Agenda Working Group (SAWG)	(i) Use TPPs developed by the product-specific working groups as framework for (ii) Characterizing the MPT pipeline from discovery through regulatory approval (iii) Prioritizing promising candidates	(i) Agenda-driven conference calls to review successive iterations of TPPs and survey responses (ii) Convening of experts charged with critiquing and debating SAWG draft to formally review SAWG recommendations	(i) Respondents to MPT Product Profiles Survey (ii) 35 experts from pharmaceutical companies, academic institutions, national regulatory authorities, global drug delivery	(i) SAWG recommendations and priorities endorsed (ii) Feedback and recommendations regarding challenges, risks, and strategies to be considered
	(iv) Analyzing overall pipeline status and gaps (v) Exploring associated regulatory implications		Efforts, and countries with greatest need for MPT products (Product Prioritization Stakeholder Meeting, October 2012)	

Reports for the starred activities are available at http://www.cami-health.org [10–13].

**Table 2:** TPP parameters for prioritizing MPT development.

Parameter	Preferred criteria	Minimally acceptable criteria
Indications	HIV + contraception (high emphasis for sub-Saharan African markets) (high emphasis for sub-Saharan African markets)	HIV + HSV (high emphasis for non-LDC markets) contraception + STI (high emphasis for Indian and Chinese markets) BV, HPV, and TV (moderate emphasis) GC + syphilis (minimal emphasis)
Route of administration	Vaginal rings	Oral pills, injectables
Dosage form and schedule	Sustained release (1–12 months) Pericoital Fast-acting Topical (vaginal)	Daily Oral
Efficacy:		
(i) HIV	80%	40%–70%
(ii) Contraception	>Current levels per contraceptive of >90%	Current levels with recommended use
(iii) STI	>80%	40%
Storage conditions	>40°C/75% RH	15–30°C/65% RH for topical/pills Refrigeration at 4°C for injectables
Shelf life	>36 months	24 months
Yearly product cost/user	<US$ 50	<US$ 100
Disposal/waste	Concealable, biodegradable user disposal	Controlled disposal (to include all associated materials (implant, injectables))
Adherence	>80% of users follow prescribed regimen	>60% of users follow prescribed regimen
Time to licensure	5 years	8–12 years (by 2020)
Reversibility	0–24 hours for oral, topical, sustained-release methods 14 days for implants, injectables	14–30 days for oral, topical, sustained-release methods 90 days for implants, injectables

**Table 3:** Multipurpose RH Vaccine Working Group: active immunization concepts.

Indication and mechanism	Immunogen, adjuvant, and delivery mode
HIV-1, HPV Stimulation of humoral and cellular immune response	DNA systemic (IM); subunit mucosal (intranasal, sublingual, and vaginal), CM cellulose (mucoadhesive)

HIV-1, HSV-2, and HPV Targeted induction of broadly neutralizing antibodies (systemic)	Synthesized and chemically modified peptide; Advax adjuvant; injected liquid

HSV, HPV, and HIV Maintain protective concentrations of cervicovaginal antibodies and/or detectable pathogen specific T-cells	Intravaginal tampon delivery of a nanoemulsion vaccine containing recombinant HSV-2 glycoprotein D and recombinant HPV 16 and 18 L1 protein and HIV glycoprotein 120

HSV, HIV Sustained protective levels of antibody and cell-mediated immunity	Subunit trimeric gp140 and HSV gD; versatile adjuvant system (PLA-NPs), systemic liquid formulation, and mucoadhesive gel carrying both antigens and immunostimulatory molecules to the same dendritic cell (prevents systemic inflammatory responses)

HPV, HBV Systemic and mucosal neutralizing antibodies	Virus-like particle (VLP) subunits, MPL or aloe-derivative adjuvant, nasal prime/boost (systemic prime/nasal boost)

HSV, HIV, and HPV Systemic and mucosal immune responses	DNA or subunit prime with HPV VLPs, gD, gp120 (intramuscular); lactococcus cocktail expressing gD, HPV E6/E7, HIV gag for mucosal boost (tablet)

HPV, sperm (immunocontraceptive can be provided separately); antibodies in fallopian tubes and in cervicovaginal mucus plus systemic antibodies and cell-mediated immunity	Salmonella vectored subunits: (a) L1 capsomeres (possibly with L2 peptide), (b) cocktail of sperm antigens; oral tablet

HIV, HSV	Codelivery of immunogens (trimeric gp140 boosts following DNA prime), and microbicides (1% tenofovir or dapivirine) via an intravaginal ring. Mucosal adjuvant is R848 (a TLR 7/8 agonist) to sustain mucosal memory

Dual-purpose HPV (multiple types) vaccine plus griffithsin microbicide (HIV, HSV)	L2 epitope fusion with griffithsin (immunogen/adjuvant); intravaginal ring (or PVA film) for burst release of HPV vaccine (L2-griffithsin fusion protein) and sustained release of griffithsin as a microbicide

HSV, HIV Systemic and mucosal protective concentrations of neutralizing antibodies	gD/Fc fusion protein, gp41 anti-idiotype; nasal prime delivered with dry inhaler; cervicovaginal boost delivered as film; FcRn-mediated transport across epithelium

**Table 4:** Reproductive Health Consensus Target Product Profile for MPT Vaccines.

Parameter	Optimally preferred
Indication and mechanism	HSV, HIV, HPV Systemic and mucosal protective concentrations of neutralizing antibodies (and cell-mediated immunity)
Target population	Women/girls: developed and developing regions
Immunogen, adjuvant, and delivery modes	Well-characterized immunogens (but range of adjuvants and delivery modes
User-action	Pharmacy or self-administered boosts
Boost schedule	Mucosal boost schedule uncertain
Typical use efficacy	HSV (70–90%); HIV (70–90%); HPV (>95%)
Side effect profile	Minimal
Additional benefits	Versatile production platform
Shelf life	Years
Storage needs	No cold chain required
Price	$1/dose
Infrastructure	Pharmacy

**Table 5:** Understanding Regional Needs and Priorities for MPT Development*.

Region	Epidemiology	Priorities, opportunities, challenges for MPT development
Sub-Saharan Africa (SSA)	*Contraception* (i) SSA lags behind global trends for increasing contraceptive prevalence and fertility decline, with high rates of unintended pregnancy and maternal mortality (ii) The region is not homogeneous: faster evolution in Southern and Eastern Africa. Demand for contraception now reaching 50% of married women, but only accessible to 20% [14–16] (iii) Method preferences also vary: unmarried women throughout SSA mainly rely on male-controlled condoms; pills, and injectables used predominantly by married women, rarely with additional protection against STIs [17]. Female condom is underutilized and comparatively expensive, with 50 million distributed annually by UNFPA [18] *STI protection* (i) Women on HIV antiretroviral therapy often do not use modern contraception, or forgo additional condom use [19]. In southern and eastern Africa, where a sizable proportion of HIV-positive women use injectable hormonal contraception [20] (ii) Syndromic management has reduced rates of bacterial STIs such as syphilis and chancroid [21], rates of the other dominant viral (STIs, HSV, HPV) extremely high, with prevalence rates up to 70% for HSV [4], between 20 and 33% HPV [22] in some cohorts (iii) Prevalence of bacterial vaginosis (BV), associated with increased risk of HIV-1 acquisition, reaches rates of 16–50% of women [23–25].	*Priorities* (i) STI prevention targets dictated by prevalence, that is, HIV, HSV, BV, trichomonas vaginalis (TV), and HPV. (ii) Strong regional preference for injectable products, but barrier methods and vaginal products also highly acceptable. *Opportunities* (i) Contraceptive uptake could increase by expanding method mix, moving toward low-dose hormonal products (and IUDs), addressing health concerns through expanded user education, focusing on populations with a high unmet need (ii) Increasing pressures for integration of services for HIV prevention, testing, PMTCT and care, and family planning services *Challenges* (i) Method needs differ for married and unmarried women, and preference varies across the region (ii) MPTs with and without a contraceptive component required for women at risk for STIs and wishing to become pregnant (iii) Given current popularity of injectable contraceptives, concern about impact of progestins on HIV acquisition is high [26]. (iv) Health interventions in low-resource and middle-income countries often experience slow uptake, necessitating interventions with long-term horizons

India	*Contraception* (i) Population growth main concern, but total fertility rate showed dramatic decline over last few decades, to total fertility rate of 2.6 [15, 27]. Decreasing significant rate of unintended pregnancies [28] instrumental in reaching replacement level fertility, a critical requirement to prevent doubling current population within next 50 years [15, 29] (ii) Only 7% of sexually active young women have ever used condoms for premarital sex; 25% of women are pregnant or mothers by age 18 [30] (iii) Current contraceptive prevalence just under 50% [15]; method mix consists primarily of female sterilization, IUDs, male condoms, and oral contraceptives; injectables and female condoms rarely used [31] *STI protection* (i) HIV prevalence in India estimated at 0.31% (2.39 million people), concentrated in high-risk groups (female sex workers, migrant workers, men who have sex with men, intravenous drug users) (ii) HIV acquisition is primarily through heterosexual sex and 39% of all infections occur in women [32] (iii) Bacterial STI prevalences overall below 10%; candidiasis and HSV-2 reach low double digits; bacterial vaginosis reported as high as 63% [33] (iv) Regions with low HIV prevalence (e.g., Bihar, Orissa, Uttar Pradesh) also have low rates for other STIs, but lead national statistics with the worst maternal mortality rates, highest fertility rates, and lowest rates of use of modern contraceptive methods [34]	*Priorities* (i) The Indian market for MPTs would be driven by priority for a contraceptive indication *Challenges* (i) Cultural factors such as women's often limited ability to act as decision-maker for own health, husband's support (as well as varying comfort levels with administration of vaginal products) will all influence uptake of MPTs

China	*Contraception* (i) At almost 85% of married women, one of world's highest rates of contraceptive use [35] (ii) Contraceptive method mix dominated by IUDs (40%) and female sterilization (almost 30%); condom use has increased with urbanization and increased income (iii) Availability of oral contraception, implants, and injectables still limited, partly due to lack of government funding and substantial regulatory approval processes, discouraging to private enterprise *STI protection* (i) Overall HIV prevalence low, almost 80% concentrated in Guangzi, Guangdong, Henan, Sichuan, Xinjian, Yunnan provinces; overall HIV prevalence estimated at 780,000, with 48,000 new HIV infections in 2011 (ii) More than 75% of HIV transmission is heterosexual; 28.6% of all HIV infections in China are in women. In economically developed provinces, for example, Dongguan, Guangdong, many new HIV cases are among migrant workers [36, 37] (iii) Due to expanded reproductive health care in government facilities, STI prevalence in China declined over past two decades, but prevalence in underserved rural areas remains high [38] (iv) STI epidemic in China is changing: while gonorrhea and HPV were main infections in past decades, nongonorrheal urethritis (NGU) and syphilis surged over last 20 years (v) Today, syphilis is the dominant STI, mainly among young migrant workers and female sex workers in richer coastal regions; chlamydia, gonorrhea, HPV, non-gonococcal urethritis (NGU), and HPV are widely distributed [39–41]	*Priorities* (i) MPT development will be driven by the need to respond to the STI epidemic, including HIV, as well as by expansion of contraceptive method mix toward a larger proportion of short-term methods

Developed Countries	*Contraception* (i) Nearly half of all pregnancies in 29 US states are unintended [42], especially in young women age 15–19 (over 80%) [43]: more than half of American women experience an unintended pregnancy, and 30% undergo an abortion [44] (ii) While unintended pregnancy rates have improved overall, socioeconomic disparities remain. Between 1994 and 2006, rate of unintended pregnancy among US higher-income women fell by 29%, while that rate among lower-income women rose by 50% [45]. Even though national teen pregnancy rate is now the lowest in 40 years, rates among Hispanic and black teens are 2 to 3 times higher than those of non-Hispanic white teens [46] *STI protection* (i) In 2007, CDC reported 1.1 million cases of chlamydia (3-fold in women compared to men), and 356,000 cases of gonorrhea (5-fold among women age 15–24 compared to women overall) [47] (ii) CDC estimates that 20% of adolescents and adults have had a genital herpes infection [48] and about 7.4 million new cases of trichomoniasis occur each year [49] (iii) Despite regulatory approval and availability of HPV vaccines, HPV continues to infect 6.2 million Americans each year [50]. (iv) Each year about 47,000 new HIV infections occur in the US [51]. In 2010, women accounted for 23% of all diagnoses and for growing majority of all heterosexual transmissions [51]; in 2010, black women accounted for 64% of new AIDS diagnoses among women, Latinas for 17%, a rate 22 times and 5 times higher, respectively, than for white women [52]	*Priorities* (i) Developed countries were found to place highest emphasis on MPTs that would serve both as contraception and be active against selected STIs, notably HSV and HPV. (ii) HIV largely seen as issue for specific subpopulations
	(v) Approximately 20% of US HIV-positive individuals unaware of their status [51, 53] (vi) HIV and high rates of other STIs burden many European countries. In 2009, almost 344,000 cases of chlamydia and almost 30,000 cases of gonorrhea reported from EU/EEA Member States [54] (vii) Chlamydia affects more women than men, and both chlamydia and gonorrhea disproportionately affect young people in this region, where 15–24 year-olds account for 75% and 40% of reported infections, respectively (viii) In 2010, close to 120,000 cases of HIV were reported by 51 European countries, 76% of those in the East [54, 55], with heterosexual contact remaining a main route of transmission, at 43% of reported HIV cases [55]	

*Table based on literature review and presentations, discussion, and analysis at January 2012 Global Forum on MPTs hosted by the Wellcome Trust [56] and Microbicides 2012 Conference.
